# Inhibition promotes long-term potentiation at cerebellar excitatory synapses

**DOI:** 10.1038/srep33561

**Published:** 2016-09-19

**Authors:** F. Binda, K. Dorgans, S. Reibel, K. Sakimura, M. Kano, B. Poulain, P. Isope

**Affiliations:** 1 Institute of Cellular and Integrative Neurosciences, CNRS, 5 Rue Blaise Pascal 67084 Strasbourg, France; 2 Chronobiotron UMS 3415, 5 Rue Blaise Pascal 67084 Strasbourg, France; 3 Department of Cellular Neurobiology, Brain Research Institute, Niigata University, Niigata 951-8585, Japan; 4 Department of Neurophysiology, Graduate School of Medicine, The University of Tokyo, Tokyo 113-0033, Japan; 5 University of Strasbourg, 5 Rue Blaise Pascal 67084 Strasbourg, France

## Abstract

The ability of the cerebellar cortex to learn from experience ensures the accuracy of movements and reflex adaptation, processes which require long-term plasticity at granule cell (GC) to Purkinje neuron (PN) excitatory synapses. PNs also receive GABAergic inhibitory inputs via GCs activation of interneurons; despite the involvement of inhibition in motor learning, its role in long-term plasticity is poorly characterized. Here we reveal a functional coupling between ionotropic GABA_A_ receptors and low threshold Ca_V_3 calcium channels in PNs that sustains calcium influx and promotes long-term potentiation (LTP) at GC to PN synapses. High frequency stimulation induces LTP at GC to PN synapses and Ca_V_3-mediated calcium influx provided that inhibition is intact; LTP is mGluR1, intracellular calcium store and Ca_V_3 dependent. LTP is impaired in Ca_V_3.1 knockout mice but it is nevertheless recovered by strengthening inhibitory transmission onto PNs; promoting a stronger hyperpolarization via GABA_A_ receptor activation leads to an enhanced availability of an alternative Purkinje-expressed Ca_V_3 isoform compensating for the lack of Ca_V_3.1 and restoring LTP. Accordingly, a stronger hyperpolarization also restores Ca_V_3-mediated calcium influx in PNs from Ca_V_3.1 knockout mice. We conclude that by favoring Ca_V_3 channels availability inhibition promotes LTP at cerebellar excitatory synapses.

In everyday life, we all benefit from the fine work performed by the cerebellum which allows us to fine tune our movements during daily actions in response to environmental changes and while executing complicate tasks such as walking or playing the violin. In line with its role in adaptive control of skilled movements and motor learning^1^,^2^,^3^,^4^ the cerebellum receives vestibular, sensory and motor information which are conveyed from the entire body to the cerebellar cortex where they converge to Purkinje neurons (PNs). PNs are the sole output of the cerebellar cortex and they receive two main excitatory inputs, parallel fibers (PFs) and climbing fibers (CFs). PFs are the axons of granule cells (GCs) which relay proprioceptive, somatosensory and vestibular information reaching the cerebellum via mossy fibers (MFs) originating from several pre-cerebellar nuclei in the brainstem and spinal cord (Fig. 1a).

Long-term plasticity has been described at GC to PN synapses with long-term depression (LTD) caused by co-activation of PFs and CF^5^ while the sole PFs stimulation leads to long-term potentiation (LTP)^6^,^7^,^8^,^9^,^10^. Furthermore, long lasting alterations in the strength of transmission at these excitatory synapses have been proposed as the molecular basis of cerebellar motor learning^1^,^11^. Indeed, mouse models lacking key molecules for LTD and LTP induction also show impairment in adaptation of the vestibulo-ocular reflex (VOR), a well- established model for cerebellum-mediated motor learning^10^,^12^,^13^,^14^,^15^.

The same bundle of PFs which excites PNs also drives molecular layer interneurons (MLIs: basket and stellate cells, Fig. 1a) which provide feedforward inhibition (FFI) through GABAergic inhibitory synapses on the same PN. PFs-mediated excitation in PNs is therefore balanced by inhibition which ultimately influences the final output of the cerebellum. Indeed, a precise time window for PFs excitatory inputs summation and spike generation in PNs is defined by FFI^16^. Sustained PFs activation also promotes repetitive MLIs stimulation and therefore FFI-mediated influence on long-term plasticity is also expected. Accordingly, the block of inhibition promotes LTD^17^ and LTP is modulated by activation of the ionotropic GABA_A_ receptor *in vivo*^18^. In addition, mice selectively lacking synaptic GABA_A_ receptors in PNs show impairment of consolidation of the vestibulo-cerebellar motor learning^19^.

How inhibition modulates long-term plasticity at PF to PN synapses is nevertheless still unknown. PNs express low threshold voltage-gated T-type calcium channels (Ca_V_3) which have been recently linked to calcium signaling, LTP at PF to PN synapses and to some aspects of motor learning^15^,^20^,^21^. Ca_V_3 channels are opened by small depolarization of the cell membrane and they generate a transient current which rapidly inactivates^22^. Recovery from inactivation requires cell membrane re-hyperpolarization and therefore Ca_V_3 channels can be influenced by inhibitory inputs as shown in thalamic neurons where GABAergic transmission promotes T-type channels-mediated low threshold calcium spikes^23^,^24^. By investigating the molecular pathway leading to LTP in PNs, we demonstrate a tight cooperation between ionotropic GABA receptors and low threshold voltage-gated T-type calcium channels (Ca_V_3) which promotes LTP at PF to PN synapses. We show that potentiation of transmission at PF to PN synapses following high frequency PFs stimulation requires calcium influx via Ca_V_3 channels and GABA_A_ receptor activation. Also, we provide evidence supporting Ca_V_3 modulation by inhibitory inputs leading to an increase in channels availability. We then conclude that FFI can control LTP at PF to PN synapses.

## Results

### LTP at PF to PN synapses is GABA_A_ receptor-dependent

**Figure 1:**
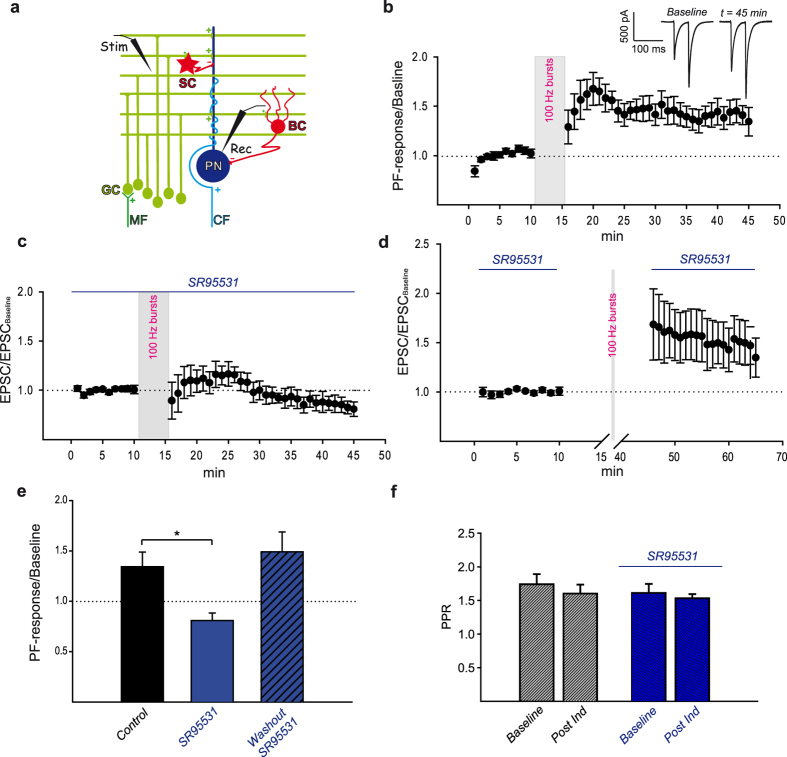
High frequency PFs stimulation induces GABA_A_ receptor dependent LTP at PF to PN synapses. A schematic representation of the cerebellar microcircuit and experimental setting is shown in panel **a**. Purkinje neurons (PN) receive excitatory inputs from climbing fibers (CF) and mossy fibers (MF) via granule cells (GC) activation. Parallel fibers excitatory synapses (green+) drive PNs and inhibitory (red −) molecular layer interneurons stellate (SC) and basket (BC) cells. Traces elicited by paired-pulses PFs stimulation (Stim, **a**) in a voltage-clamped PN (Rec, **a**) at different time points are showed in the inset of panel **b**. The *Baseline* trace was obtained from averaging of all recordings during baseline while the *t* = *45* *min* trace is the average of three consecutive PF-mediated responses recorded every 20 seconds at the indicated time point. High frequency PFs stimulation induced a long lasting increase in PNs response (MLI_dep_-LTP, **b**: mean ± SEM, N = 5, RM ANOVA P < 0.001). Bath application of the GABA_A_ receptor antagonist SR95531 prevented MLI_dep_-LTP (**c**, mean ± SEM, N = 5, RM ANOVA P < 0.001). A summary graph for SR95531-mediated effect on MLI_dep_-LTP is shown in panel **e** (bar represents normalized PF-Rsp at t = 45 min, mean ± SEM, data from panel b and c), *indicates a statistically significant difference among values (t-test, P = 0.011). Keeping GABAergic transmission intact only during high frequency PFs stimulation was sufficient to induce MLI_dep_-LTP (**d**). For these experiments, a 10 minutes baseline was established with SR95531 and the antagonist was washed out for at least 15 minutes before the induction protocol was applied; SR95531 was added back to the recording chamber immediately or 15 minutes after high frequency stimulation (**d**, mean ± SEM, N = 7, RM ANOVA P < 0.001; **e**, bar represents normalized PF-Rsp at t = 65 min, mean ± SEM, data from panel d). PPRs value (mean ± SEM) for the baseline (t = 10) and the post induction phase (t = 45) with or without bath application of SR95531 are shown in panel **f**.

The role played by inhibition in LTP induction at PF to PN synapses was investigated in acute cerebellar slices. In voltage clamped PNs (V_h_ = −60 mV), molecular layer electrical paired-pulses stimulation (20 Hz) induced PF-mediated fast inward currents with facilitation at the second response (Fig. 1b inset, Baseline). The induced response (PF-Rsp) was stable within 10 minutes from the beginning of the recording (Fig. 1b, average baseline value: −781.31 ± 25.77 pA, mean ± SEM, N = 5). Once baseline was established, PFs were stimulated at high frequency (burst of 15 pulses at 100 Hz repeated every 3 seconds for a period of 5 minutes) while PNs were switched to current clamp mode. This induction protocol was chosen to mimic GCs physiological activity^25^,^26^,^27^ and to ensure reliable PF to MLI transmission whose failure rate has been shown to decrease at high frequency rate^28^. Train of PFs bursts stimulation caused a long lasting potentiation of the PFs-induced response (Fig. 1b). Higher values were reached at an early phase (Fig. 1b, normalized PF-Rsp_t20_ = 1.68 ± 0.18, mean ± SEM, N = 5) with a later stable lower level maintained until the end of the recording (steady state) (Fig. 1b inset, t = 45 min; Fig. 1b, normalized PF-Rsp_t45_ = 1.34 ± 0.14, mean ± SEM, N = 5). Since LTP can be expressed at presynaptic and/or postsynaptic site^29^, the paired-pulse ratio (PPR) was investigated to discriminate between these possibilities. PPRs at steady state showed no statistically significant difference when compared to baseline (Fig. 1f, PPR_t10_ = 1.74 ± 0.15, PPR_t45_ = 1.6 ± 0.13, mean ± SEM; PPR_t10_ vs PPR_t45_: one-way repeated measures (RM) ANOVA, Tukey’s post hoc test, P > 0.05, N = 5) indicating that a change in the presynaptic probability of release was unlikely.

The role of GABAergic transmission in LTP induction was then pharmacologically investigated by bath application of the selective GABA_A_ receptor antagonist SR95531 (5 μM). PNs holding current (I_holding_) and baseline PF-mediated response decay time constant (τ_off_) displayed no statistically significant difference in the presence of the antagonist (I_holding_ SR95531: −346.74 ± 44.44 pA, mean ± SEM, I_holding_ control: −241.63 ± 55.46, mean ± SEM, P = 0.177, t test, N = 5; τ_off_ SR95531: 7.14 ± 0.8 ms, mean ± SEM, τ_off_ control: 5.49 ± 2 ms, mean ± SEM, P = 0.467, t-test, N = 5) suggesting that, under our experimental conditions, PNs were clamped at a membrane potential (V_h_ = −60 mV) close to the equilibrium potential of GABA_A_ receptor-mediated currents (calculated E_Cl_ = −63 mV) and that the PF-mediated response was mostly mediated by AMPA receptors.

Blocking the GABA_A_ receptor had a profound effect on LTP (Fig. 1e) which was completely abolished by SR95531 (Fig. 1c, t = 45, normalized EPSC = 0.81 ± 0.07, mean ± SEM, N = 5; Fig. 1e, mean ± SEM, t-test, P = 0.011) with no statistically significant change in PPRs (Fig. 1f, PPR_t10_ = 1.61 ± 0.13, PPR_t45_ = 1.53 ± 0.06, mean ± SEM; PPR_t10_ vs PPR_t45_: one-way repeated measures (RM) ANOVA, Tukey’s post hoc test, P > 0.05, N = 5).

LTP at PF to PN synapses could therefore be induced by high frequency PFs stimulation provided that the ionotropic GABAergic transmission was intact, supporting a role of MLIs in long-term potentiation at PF to PN synapses; for simplicity we will refer to LTP established under our experimental condition as MLI dependent LTP (MLI_dep_-LTP) in the later part of the text.

PF to MLI synapses and the MLIs network can also be affected by long-term plasticity that could ultimately influence our recordings^30^,^31^,^32^,^33^,^34^. We therefore isolated the effect of high frequency PFs stimulation on EPSCs by applying SR95531 during the baseline and the post induction phase while maintaining inhibition active during the induction protocol. Keeping inhibitory transmission intact while applying PFs bursts stimulation was necessary and sufficient to establish MLI_dep_-LTP at PF to PN synapses (Fig. 1d) with no statistically significant change in PPRs (PPR_t10_ = 1.78 ± 0.08, PPR_t65_ = 1.58 ± 0.1, mean ± SEM; PPR_t10_ vs, PPR_t65_, RM ANOVA, Tukey’s post hoc test, P = 0.054, N = 7). Furthermore, EPSC increase was comparable to the potentiation obtained with intact GABAergic transmission (Fig. 1e,d: normalized EPSC_t65_ = 1.49 ± 0.2; Fig. 1e,b: normalized PF-response_t45_ = 1.34 ± 0.14, P = 0.593, t-test).

Taken together our data support the requirement of GABA_A_ receptor activation in MLI_dep_-LTP; MLI_dep_-LTP is likely postsynaptic and it is expressed at PF to PN synapses.

### Molecular pathway to MLI_dep_-LTP

**Figure 2:**
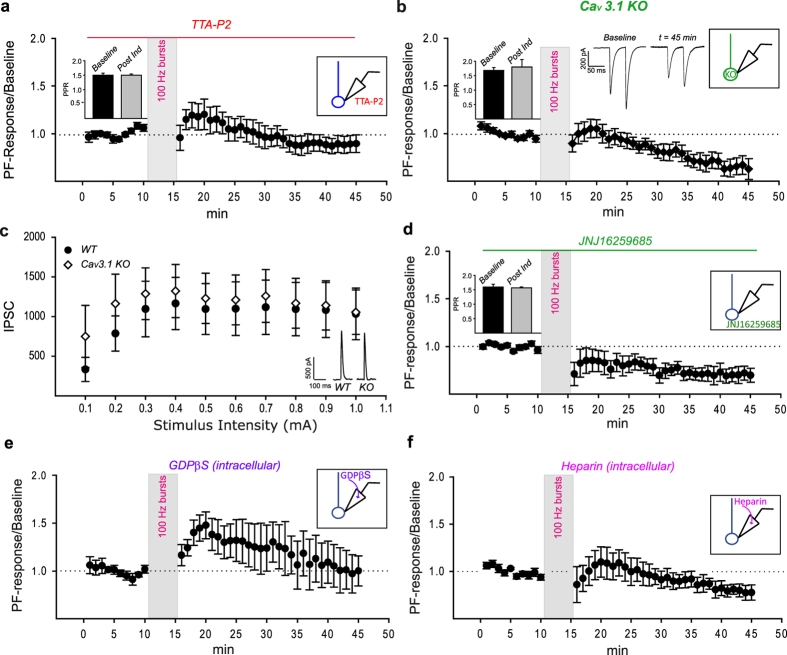
Molecular pathway to MLI_dep_-LTP. MLI_dep_-LTP was effectively prevented by the broad spectrum low threshold voltage-gated T-type calcium channels specific antagonist TTA-P2 (**a**, mean ± SEM, N = 5, RM ANOVA P < 0.001). To identify the Ca_V_3 isoform required for the GABA-dependent potentiation, long-term plasticity experiments were performed in Ca_V_3.1 KO mice. MLI_dep_-LTP is absent in Ca_V_3.1 KO mice (**b**, mean ± SEM, N = 5, RM ANOVA P < 0.001) under the same experimental condition that leads to GABA_A_ receptor-dependent potentiation of PF to PN transmission in WT mice and traces from a representative experiment are shown in the middle inset. PF-induced inhibitory responses were recorded in PNs from WT and Ca_V_3.1 KO mice (**c**, inset) and input/output curves obtained (**c**: WT, black circle: N = 8; KO, white diamond: N = 10, mean ± SEM). No statistically significant difference was detected among the two curves (P > 0.05, t-test at all stimulus intensities). MLI_dep_-LTP also depends on mGluR1 and intracellular calcium stores; high frequency PFs stimulation failed to induce MLI_dep_-LTP when activation of the metabotropic glutamate receptor mGluR1was prevented by bath application of the specific antagonist JNJ16259685 (**d**, mean ± SEM, N = 5, RM ANOVA P < 0.001). Inclusion of the non-hydrolysable GDP analog GDPβS (**e**, mean ± SEM, N = 3) or heparin (**f**, mean ± SEM, N = 6, RM ANOVA P = 0.006) in the intracellular recording solution also impaired MLI_dep_-LTP. PPRs value (mean ± SEM) for the baseline (t = 10 min) and the post induction phase (t = 45 min) under each condition are shown in the left insets in panel **a**,**b**,**d**.

Low threshold voltage-gated Ca_V_3 channels have recently been linked to postsynaptic LTP at PF to PN synapses^15^ and they are influenced by inhibitory inputs as shown in thalamic and deep cerebellar nuclei (DCN) neurons^23^,^24^,^35^,^36^,^37^. We therefore investigated their possible role in MLI_dep_-LTP by pharmacologically blocking their activation with the highly specific T-type calcium channels antagonist TTA-P2^38^ (Merck; 500 nM). In the presence of the Ca_V_3 channels antagonist, high frequency PFs stimulation failed to induce MLI_dep_-LTP with the induced responses slightly decreasing below baseline level after the first transient increment (Fig. 2a, t = 45, normalized PF-Rsp = 0.9 ± 0.09 mean ± SEM, N = 5); PPRs showed no statistically significant difference from baseline (Fig. 2a inset, PPR_t10_ = 1.5 ± 0.072, PPR_t45_ = 1.5 ± 0.04; mean ± SEM; PPR_t10_ vs PPR_t45_, RM ANOVA, Tukey’s post hoc test, P > 0.05, N = 5).

All three Ca_V_3 isoforms are found in PNs with weak Ca_V_3.2 channels staining^39^ and more pronounced Ca_V_3.1 and Ca_V_3.3 channels expression^39^,^40^. PFs bursts-induced calcium transients in mature PNs are mostly mediated by Ca_V_3.1 channels^21^ and Ca_V_3.1 KO mice showed LTP impairment^15^. We therefore investigated whether Ca_V_3.1 channels play a role in MLI_dep_-LTP in acute cerebellar slices from Ca_V_3.1 KO mice^41^. Under the same experimental condition which led to MLI_dep_-LTP in WT mice, Ca_V_3.1 KO mice showed no MLI_dep_-LTP (Fig. 2b, N = 5). Also, PPRs value showed no statistically significant difference to baseline (Fig. 2b inset, PPR_t10_ = 1.68 ± 0.1, PPR_t45_ = 1.8 ± 0.26, mean ± SEM; PPR_t10_ vs PPR_t45_, RM ANOVA, Tukey’s post hoc test, P > 0.05, N = 5).

In order to exclude any effect of Ca_V_3.1 absence on MLI to PN transmission that could potentially interfere with MLI_dep_-LTP induction, the total PF-induced inhibition was measured in WT and Ca_V_3.1 KO mice. Input/output curves showed no significant difference in Ca_V_3.1 KO mice compared to WT animals (Fig. 2c, minimum stimulus intensity: WT = 382.88 ± 152.41 pA, N = 8; KO = 748.95 ± 391.01 pA, N = 10, P = 0.379, t-test) supporting a normal MLI to PN transmission. The absence of MLI_dep_-LTP is therefore a direct consequence of impaired calcium influx in Ca_V_3.1 KO PNs dendrites^15^ rather than a secondary effect due to altered inhibition.

A functional coupling between mGluR1 receptors and Ca_V_3.1 channels has been identified in PNs with the activation of the G-coupled receptor leading to the potentiation of calcium influx through T-type calcium channels^21^ and therefore mGluR1activation may be required for the described long-term potentiation. Indeed, blocking mGluR1 by bath application of the specific antagonist JNJ16259685 (2 μM) prevented MLI_dep_-LTP (Fig. 2d, N = 5) with no statistically significant change in PPRs when compared to baseline (Fig. 2d inset, PPR_t10_ = 1.59 ± 0.09, PPR_t45_ = 1.57 ± 0.03, mean ± SEM, N = 5; PPR_t10_ vs PPR_t45_ P > 0.05, RM ANOVA, Tukey’s test). Strikingly, blocking the receptor also revealed a mGluR1-independent LTD (normalized PF-Rsp_t45_ = 0.7 ± 0.07, normalized PF-Rsp_t1_ = 1 ± 0.02, P = 0.025 RM ANOVA, Tukey’s post hoc test). As expected by MLI_dep_-LTP impairment caused by mGluR1 inactivation in PNs, the inclusion of the non-hydrolysable GDP analog GDPβS (2 mM) in the patch pipette also interfered with long term plasticity (Fig. 2e, N = 3). mGluR1 activation induces release of calcium from intracellular stores which could therefore be implicated in MLI_dep_-LTP. When calcium release from intracellular store via IP3 receptors was prevented by inclusion of heparin (50 μg/ml) in the patch pipette^42^, MLI_dep_-LTP was also impaired (Fig. 2f, N = 6) suggesting that calcium influx via Ca_V_3 channels and internal stores might cooperate for MLI_dep_-LTP induction.

These experiments support the requirement of a postsynaptic molecular cascade for the expression of MLI_dep_-LTP at PF to PN synapses; we showed that MLI_dep_-LTP is Ca_V_3 channels, mGluR1 and internal calcium stores dependent.

### Ionotropic GABA receptors and Ca_V_3 channels cooperation is required for MLI_dep_-LTP

**Figure 3:**
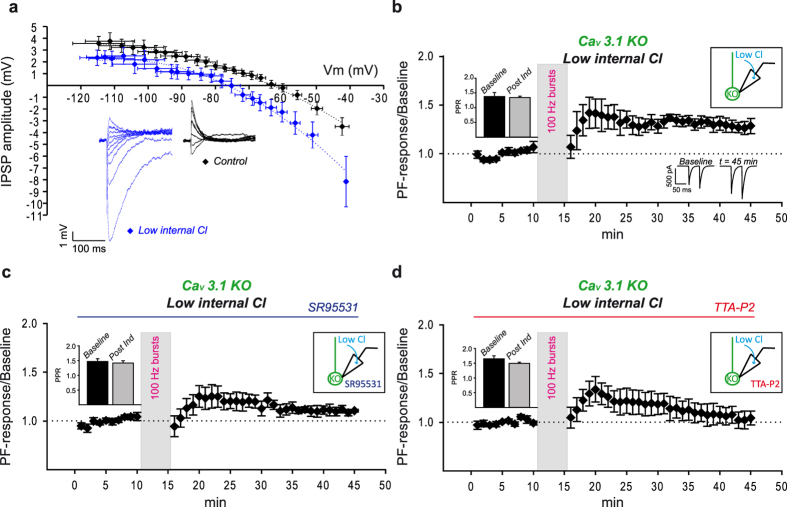
MLI_dep_-LTP is Ca_V_3.1 dependent and it relies on GABA_A_ receptor and T-type calcium channels cooperation. Change in intracellular chloride concentration affects IPSPs/V_m_ curves in PNs from Ca_V_3.1 KO mice. Lowering intracellular chloride caused a 13 mV hyperpolarizing shift of IPSP/Vm curves (**a**, blue diamond, N = 7, mean ± SEM) compared to control condition (**a**, black diamond, N = 7, mean ± SEM). IPSPs recorded at different V_m_ from two representative PNs are shown in the inset of panel **a** in control (black) and low chloride (blue) condition. Dot lines represent the polynomial fit of IPSP/Vm curves obtained under the two experimental conditions. MLI_dep_-LTP was rescued when the low internal chloride recording solution was used (**b**, mean ± SEM, N = 5, RM ANOVA P < 0.001) and traces from one exemplificative experiment are shown in the inset at the bottom of the panel. The rescued MLI_dep_-LTP was dependent on GABA_A_ receptor (**c**: mean ± SEM, N = 5, RM ANOVA P < 0.001) and T-type calcium channels (**d**: mean ± SEM, N = 5, RM ANOVA P < 0.001) activation. PPRs value (mean ± SEM) for the baseline (t = 10 min) and the post induction phase (t = 45 min) under each condition are showed in the left insets in panel **c**,**d**.

Ca_V_3 channels open in response to small depolarization of the cell, quickly inactivate and recovery from inactive state depends on cell membrane re-hyperpolarization after channel opening^22^. The three Ca_V_3 channel isoforms are characterized by different activation curves with Ca_V_3.1 (half activation V_a_: −60 ± 0.9 mV at 37 °C) and Ca_V_3.2 channels (half activation V_a_: −51.5 ± 1 mV at 37 °C) opening at more depolarized membrane potential compared to Ca_V_3.3 channels (half activation V_a_: −73.5 ± 1.3 mV at 37 °C)^43^ which therefore requires stronger hyperpolarization to de-inactivate. Modulation of GABA_A_-mediated transmission could therefore impact Ca_V_3 channels availability and affect MLI_dep_-LTP. We investigated this hypothesis by lowering the chloride concentration in the internal solution for MLI_dep_-LTP experiments in Ca_V_3.1 KO mice to obtain a stronger GABA_A_ receptor-mediated hyperpolarization in PNs as shown by the 13 mV negative shift of the IPSP reversal potential when compared to control condition (control, N = 7: −63 mV; low chloride, N = 7: −76 mV) (Fig. 3a); a stronger hyperpolarization might enhance Ca_V_3.3 channels availability and therefore improve Ca_V_3 channels-mediated intracellular calcium rise in the Ca_V_3.1 KO during bursts of PFs stimulation and facilitate MLI_dep_-LTP induction. Indeed, under these experimental conditions, high frequency PFs stimulation generated a long lasting increase of transmission in Ca_V_3.1 KO mice (Fig. 3b, t = 45, normalized PF-Rsp = 1.29 ± 0.17, mean ± SEM, N = 5). The recovery of LTP depended on GABA_A_ activation (KO-MLI_dep_-LTP) since it was absent in presence of SR95531 (Fig. 3c, normalized EPSC_t10_ = 1.11 ± 0.01, normalized EPSC_t45_ = 1.05 ± 0.04, P > 0.05 RM ANOVA, Tukey’s post hoc test, N = 5). More importantly, TTA-P2 bath application (Fig. 3d) also prevented KO-MLI_dep_-LTP (normalized PF-Rsp_t45_ = 1.04 ± 0.07, normalized PF-Rsp_t10_ = 0.99 ± 0.02, P > 0.05 RM ANOVA, Tukey’s post hoc test, N = 5) indicating that the enhanced availability of an alternative PN-expressed Ca_V_3 isoform could compensate for the lack of Ca_V_3.1 channels.

Under all experimental condition tested, PPRs showed no difference when compared to baseline value (Fig. 3c inset, control: PPR_t10_ = 1.38 ± 0.13, PPR_t45_ = 1.34 ± 0.04, N = 5; Fig. 3d inset, SR95531: PPR_t10_ = 1.47 ± 0.09, PPR_t45_ = 1.42 ± 0.08, N = 5; Fig. 3e inset, TTA-P2: PPR_t10_ = 1.65 ± 0.1, PPR_t45_ = 1.49 ± 0.04, N = 5; mean ± SEM, PPRt_10_ vs PPR_t45_, P > 0.05, RM ANOVA, Tukey’s test).

### Ca_V_3 channels-mediated calcium transient is under the control of ionotropic GABA receptors

**Figure 4:**
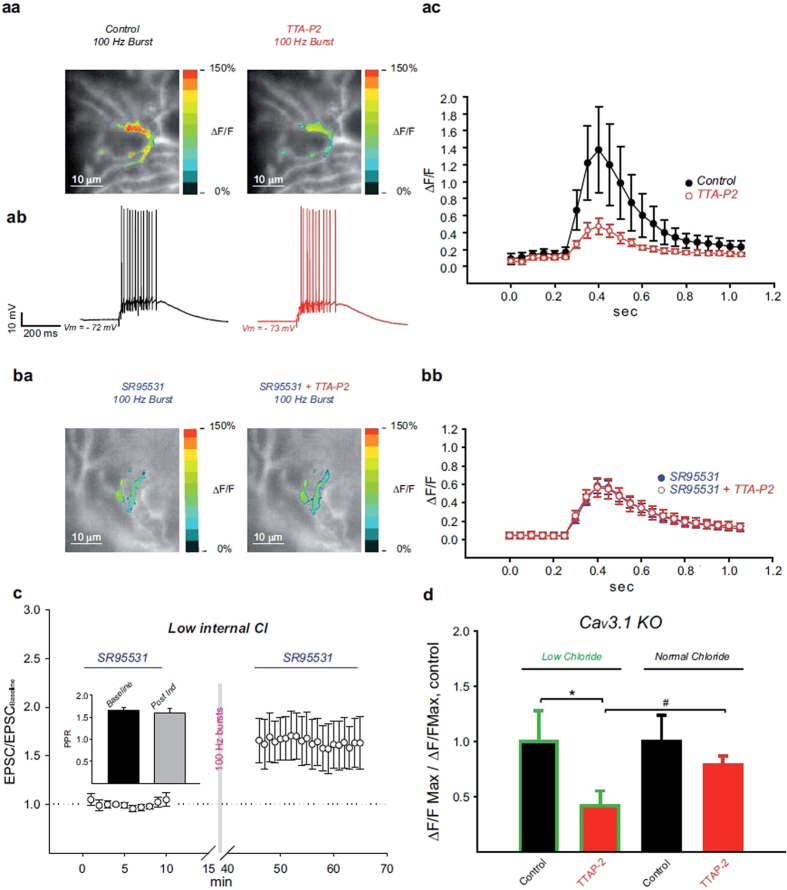
Ca_V_3-mediated calcium influx is controlled by inhibition. High frequency PFs stimulation caused depolarization (**ab**) and calcium increase in PNs (**aa**, left panel; **ba**, left panel); for each representative cell showed, the average ΔF/F signals was obtained at the peak of the response under the different experimental conditions (pseudocolor) and an it was superimposed on the image of the PN at resting state (grayscale). While TTA-P2 bath application strongly affected the calcium transient recorded in control condition (**aa**, right panel), the antagonist showed no effect in presence of SR95531 (**ba**, right panel). Quantified relative change in fluorescence (ΔF/F) showed a large TTA-P2 sensitive component revealing that calcium influx is mostly mediated by Ca_V_3 activation but only in control condition (**ac**, mean ± SEM, N = 5, ANOVA P < 0.001). Ca_V_3-mediated calcium influx is lost when inhibition was blocked by bath application of SR95531 (**bb**, mean ± SEM, N = 5, ANOVA P < 0.001). High frequency PFs stimulation in low internal chloride induced MLI_dep_-LTP (**c**, mean ± SEM, N = 6, RM ANOVA P < 0.001); PPRs (mean ± SEM) value for the baseline (t = 10 min) and the post-induction phase (t = 65 min) are shown in the panel **c** inset. The effect of different intracellular chloride concentrations on the PFs-induced calcium transient was evaluated in PNs from Ca_V_3.1 KO mice (**d**). The TTA-P2 sensitive component of the calcium transient observed in low chloride (*Low chloride*: control = 1 ± 0.23, mean ± SEM, N = 5; TTA-P2: 0.42 ± 0.13, mean ± SEM, N = 5, P = 0.017, paired t-test) was lost when the normal chloride internal solution was used (*Normal Chloride*, control: 1 ± 0.23, mean ± SEM, N = 5; TTA-P2: 0.79 ± 0.08, mean ± SEM, N = 5, P = 0.446, paired t-test). Before cell averaging, the maximal ΔF/F value obtained before and after TTA-P2 bath application for each cell was normalized to the mean ΔF/F value obtained in control condition. *statistically significant difference, paired t-test. ^#^statistically significant difference, t-test.

The recovery of MLI_dep_-LTP in Ca_V_3.1 KO mice supports a functional coupling between GABA_A_ receptors and Ca_V_3 channels important for LTP at PF to PN synapses. The influence of inhibition on Ca_V_3-mediated calcium transient elicited by high frequency PFs stimulation was therefore tested in PNs from WT mice by calcium imaging (Fig. 4). For these experiments, the calcium indicator Oregon Green BAPTA 6F (400 μM) was added to the low chloride internal solution and loaded into the recorded PN via the patch pipette. To prevent calcium release from intracellular stores heparin (50 μg/ml)^42^ was also included in the patch clamp intracellular recording solution. The variation of intracellular calcium concentration was monitored in current clamped PNs (Fig. 4aa–ba) while PFs were stimulated by a single 100 Hz burst (15 pulses). Stimulus intensity was set to induce PFs-mediated responses (Fig. 4ab) similar to those recorded during the LTP induction protocol. The raise in intracellular calcium concentration caused by high frequency PFs stimulation (Fig. 4aa, left panel) was largely Ca_V_3 channels-mediated as shown by fluorescence attenuation following TTA-P2 bath application (Fig. 4aa, right panel); the relative change in fluorescence (ΔF/F) was strongly influenced by TTA-P2 with a 64% reduction of its peak value by the antagonist (Fig. 4ac, ΔF/F_Control_ = 1.37 ± 0.5, ΔF/F_TTA-P2_ = 0.47 ± 0.09, mean ± SEM, P = 0.041, ANOVA, Bonferroni post hoc test, N = 5). Interestingly, the Ca_V_3 channels-mediated component of the calcium transient was completely lost when inhibition was blocked. In presence of SR95531 (Fig. 4ba, left panel), the induced increase in intracellular calcium was TTA-P2 insensitive (Fig. 4ba, right panel and Fig. 4bb, N = 5) and therefore mediated by high threshold voltage gated calcium channels.

Plasticity experiments were then conducted with the low chloride intracellular recording solution; when high frequency PFs stimulation was applied at low intracellular chloride concentration (Fig. 4c), MLI_dep_-LTP was slightly bigger (Fig. 4c, EPSC_/baseline_t_65_ = 1.63 ± 0.27, mean ± SEM, N = 6) compared to normal chloride (Fig. 1d, EPSC_/baseline_t_65_ = 1.49 ± 0.2, N = 7) but with no statistically significant difference (normal vs low chloride, t = 65 min, P = 0.678, t-test); PPRs showed no statistically significant difference when compared to baseline value (Fig. 4c inset, PPR_t10_ = 1.66 ± 0.07, PPR_t65_ = 1.59 ± 0.11, mean ± SEM, N = 6, P > 0.05, RM ANOVA, Tukey’s test).

These findings demonstrate that ionotropic GABA receptors activation is required for Ca_V_3 channels-mediated calcium rise in PNs during high frequency PFs stimulation.

In agreement with the recovery of MLI_dep_-LTP observed in Ca_V_3.1 KO mice, the calcium transient recorded in PNs from KO mice was largely Ca_V_3 channels-mediated only when elicited by PFs stimulation at low intracellular chloride concentration (Fig. 4d, *low chloride*); bath application of TTA-P2 strongly reduced calcium influx in low chloride (normalized ΔF/F_MAX_-_Control_ = 1 ± 0.27, normalized ΔF/F_MAX_-_TTAP2_ = 0.42 ± 0.13, mean ± SEM, N = 5, P = 0.017, paired t-test) while no statistically significant difference (normalized ΔF/F_MAX_-_Control_ = 1 ± 0.23, normalized ΔF/F_MAX_-_TTAP2_ = 0.79 ± 0.08, mean ± SEM, N = 5, P = 0.446, paired t-test) was detected in the presence of the antagonist when the regular internal solution was used (Fig. 4d, *normal chloride*).

Taken together these results support the fundamental role of Ca_V_3 channels-mediated calcium influx in MLI_dep_-LTP induction at PF to PN synapses and the requirement of GABA_A_ receptors activation to ensure the availability of Ca_V_3 channels for opening during bursts of PFs activation.

## Discussion

In the cerebellar cortex, GCs activation by MFs input leads to excitatory (monosynaptic) and inhibitory (di-synaptic) events in PNs and their interaction in the induction of LTP at PFs excitatory synapses is described in this article. We showed that high frequency PFs stimulation caused LTP at PF to PN synapses only when MLIs-mediated GABAergic transmission was intact (Fig. 1).

MLI_dep_-LTP is induced by a postsynaptic mechanism as showed by its recovery in Ca_V_3.1 KO mice (Fig. 3b) and its impairment by the intracellular block of GPCRs (Fig. 2e) and IP3 receptors (Fig. 2f); MLI_dep_-LTP is GABA_A_ receptors (Fig. 1), Ca_V_3 channels (Fig. 2a,b), mGluR1 receptors (Fig. 2d,e) and intracellular calcium store (Fig. 2f) dependent.

Ca_V_3 channels-dependent LTP has been previously reported to be induced at PF to PN synapses in the presence of the GABA_A_ receptor antagonist bicuculline^15^ in apparent discrepancy with our results. Nevertheless, PNs have been shown to express the ionotropic GABA receptor bicuculline-insensitive ρ subunits^44^,^45^ suggesting that bicuculline might not be able to completely eliminate inhibition in PNs. Indeed, we were able to record a bicuculline-insensitive IPSC component in PNs following PFs stimulation (Supplementary Information); after bath application of bicuculline (20 μM), 6% of the total IPSQs was still present (Supplementary Figure S1 panel a and panel b) and it was completely blocked by the following application of SR95531 (5 μM). The fact that LTP was successfully induced in the presence of bicuculline suggests that this residual bicuculline-insensitive component of the ionotropic GABA receptors-mediated response was sufficient to mediate Ca_V_3 channels recovery from inactivation at least in WT mice. In agreement, MLI_dep_-LTP was unaffected by bath application of bicuculline (Supplementary Figure S1 panel c and panel d).

The MLI network can be modulated by long-term plasticity and PF to MLI synapses can be potentiated^32^ at low frequency stimulation (2–8 Hz) while high frequency stimulation induces LTD^30^,^31^. Also, high frequency PFs stimulation causes LTP at inhibitory MLI to MLI synapses^34^ via increase of GABA release^33^. High frequency PFs stimulation could therefore reduce inhibitory inputs to PNs via decreased MLIs excitation and/or by increasing inhibition onto MLIs. The decreased inhibition in PNs could result in indirect potentiation of PFs-mediated responses in these neurons. No significant GABA_A_-mediated component in the PFs-mediated responses was observed under our experimental conditions and therefore, if present, indirect potentiation should have had little influence on our recordings. This was confirmed by investigating the specific impact of high frequency PFs stimulation on excitatory transmission by blocking GABAergic inputs before and after the induction protocol. Excitatory transmission at PF to PN showed postsynaptic potentiation to level comparable to what previously observed (Fig. 1d). GABA_A_ receptor activation is therefore required for MLI_dep_-LTP at PF to PN synapses only during the induction phase. This and the intracellular pharmacological block of MLI_dep_-LTP (Fig. 2e,f) strongly argue against indirect potentiation and they support involvement of inhibition in LTP induction at PF to PN synapses.

By investigating the molecular pathway leading to MLI_dep_-LTP we have provided evidence for a tight cooperation among GABAergic transmission and low threshold voltage-gated calcium channels in PNs (Figs 3 and 4). We propose an active postsynaptic role for GABAergic transmission in MLI_dep_-LTP induction with inhibition modulating T-type Ca_V_3 calcium channels availability (i.e. by favoring de-inactivation). This hypothesis is supported by the recovery of MLI_dep_-LTP in Ca_V_3.1 KO mice by the intracellular modulation of the chloride electrochemical gradient in PNs (Fig. 3b). As shown by KO- MLI_dep_-LTP dependency on TTA-P2 (Fig. 3d), an alternative Ca_V_3 channels isoform is recruited when GABA_A_ receptor activation favored hyperpolarization toward more negative potentials (Fig. 3a). Accordingly, the TTA-P2-sensitive component of the calcium transient was restored in Ca_V_3.1 KO mice when the intracellular chloride was decreased (Fig. 4d). Furthermore, Ca_V_3-mediated calcium influx and MLI_dep_-LTP was detected in PNs from WT mice only when inhibitory GABAergic transmission was intact (Figs 4a,b and 1).

MLIs-mediated postsynaptic response causes membrane potential re-hyperpolarization in depolarized PNs both in the dendritic compartment and at the soma^46^,^47^. Since high frequency PFs stimulation is able to bring PNs membrane voltage to firing threshold (Fig. 4ab), the recovery of MLI_dep_-LTP (Fig. 3b) and Ca_V_3 channels-mediated calcium transient (Fig. 4d) in Ca_V_3.1 KO mice suggests that activation of GABA_A_ receptors favor re-hyperpolarization toward a level suitable for Ca_V_3 channels de-inactivation. Ca_V_3 channels inactivation has been shown to be effectively removed by GABA_A_-mediated IPSPs in DCN neurons^35^,^36^,^37^; the IPSP reversal potential in these neurons (−75 mV)^37^ is close the one obtained under our experimental condition in low chloride (Fig. 3a) supporting an effective Ca_V_3 channels de-inactivation also in our experiments.

Ca_V_3.3 channels are highly expressed in PNs and they require a stronger hyperpolarization to recovery from inactivation; based on its expression pattern and biophysical characteristics, Ca_V_3.3 channels are therefore the isoform most likely to be involved in the rescue of MLI_dep_-LTP in Ca_V_3.1 KO mice although a role of Ca_V_3.2 channels cannot be excluded at this time^48^. The reversal potential of GABAergic currents (E_GABA_: −85/−87 mV) measured in mature PNs^49^,^50^ predict a strong hyperpolarization in PNs caused by GABA_A_ receptors activation suggesting that Ca_V_3.3 channels might be also recruited under physiological conditions and it might also participate with Ca_V_3.1 in MLI_dep_-LTP induction. Ca_V_3.3 channels sole requirement is nevertheless unlikely. Cerebellar long-term plasticity is a calcium-dependent mechanism^7^ and PFs-mediated increment of calcium in PNs spines is mostly mediated by Ca_V_3.1channels even though in Ca_V_3.1 KO mice a residual T-type dependent influx is still present^21^. Furthermore, Ca_V_3.1 channels-mediated calcium influx is potentiated by mGluR1 activation and MLI_dep_-LTP dependency on this metabotropic glutamate receptor (Fig. 2d,e) further supports the requirement for the Ca_V_3.1 channel isoform in MLI_dep_-LTP. Interestingly, the impairment exhibited by Ca_V_3.1 KO mice in long term VOR phase-reversal training seems less severe when compared to the one of WT mice systemically injected with TTA-P2^15^ suggesting that Ca_V_3.3 channels might also participate in cerebellar-mediated motor learning in Ca_V_3.1 KO mice and slightly attenuate their phenotype.

When MLI_dep_-LTP was compromised, decrease efficiency in PFs transmission was also revealed unmasking a pathway leading to depression at PF to PN synapses. Interestingly, this long term depression was still present when mGluR1 was inactivated (Fig. 2d). At PF to PN synapses, mGluR1 plays a central role in LTD^51^,^52^,^53^,^54^,^55^ but long term depression is nevertheless also reliably induced by nitric oxide (NO) uncaging when coupled to PNs depolarization^56^ suggesting that this PF-released anterograde messenger^57^ together with the depolarization-induced intracellular calcium raise in PNs might be sufficient to permanently decrease the synaptic transmission strength at these synapses. Indeed a NO synthase (NOS) dependent LTD has been previously described at PF to PN synapses^58^; this LTD requires NMDA receptors activation^58^ and it is mGluR1 independent^59^. NO is likely to be released under our experimental conditions and therefore it might play a role in the depression observed whenever MLI_dep_-LTP was impaired.

**Figure 5:**
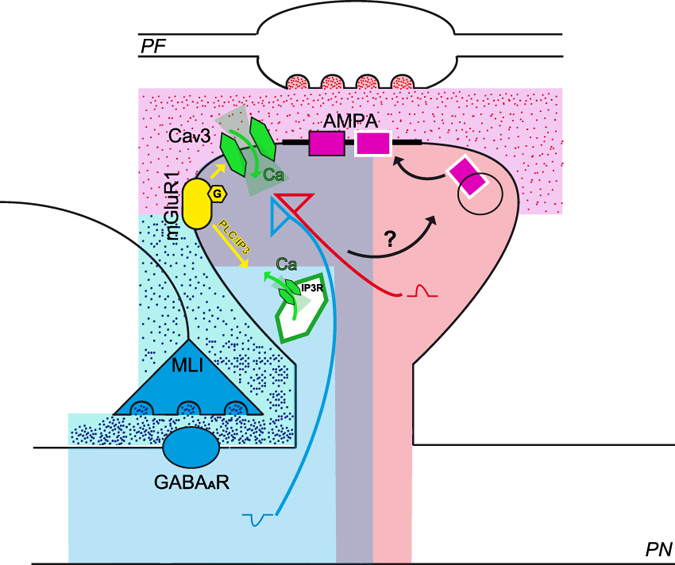
MLI_dep_-LTP model. Schematic representation of the parallel fiber (*PF*) to Purkinje neuron (*PN*) synapse with a PF varicosity drawn in the upper part and the corresponding PN spine shown below. Molecular layer interneuron inhibitory synapse (MLI) is also shown in the scheme. Following high frequency parallel fibers (PF) stimulation, GABA_A_-mediated hyperpolarization (blue shadow) limits AMPA-induced depolarization (pink shadow) to a range suitable for Ca_V_3 activation (light violet shadow). AMPA-mediated depolarization (red arrow) activates Ca_V_3 calcium channels dependently on their availability regulated by inhibition (blue arrow) and Ca_V_3-mediated calcium influx is enhanced by mGluR1 activation (yellow arrow). mGluR1 activation also leads to calcium release from intracellular stores via IP3 receptors. The described molecular steps initiate the cascade that leads to MLI_dep_-LTP and downstream events are still to be determined.

A simple model describing the first events leading to MLI_dep_-LTP at PF to PN synapses can be proposed (Fig. 5): high frequency PFs stimulation activates AMPA receptors and causes PN dendrites depolarization counterbalanced by the GABA_A_-induced hyperpolarization elicited by MLIs; membrane depolarization triggers T-type calcium channels activation and their availability for opening is controlled by inhibition. In order to induce MLI_dep_-LTP at PF to PN synapses, Ca_V_3 channels require mGluR1 activation that potentiates Ca_V_3.1 channels-mediated calcium influx via a PLC independent pathway^21^. Via the G_q_/PLC pathway, mGluR1 activation also leads to the release of calcium from the intracellular stores via activation of IP3 receptors; intracellular released calcium also seems to be required for MLI_dep_-LTP.

While high frequency stimulation of PFs induced a mGluR1-dependent LTP *in vivo*^18^, low frequency (1 Hz) stimulation of PFs in the molecular layer of cerebellar slices induced a long lasting enhancement of transmission at PF to PN synapses which was unaffected by the pharmacological inactivation of this metabotropic receptor^9^. Together with our results, these findings support the existence of two distinct pathways leading to LTP which are differently engaged by PFs activity. Low frequency stimulation induces a mGluR1 independent potentiation which is unaffected by GABAergic ionotropic transmission impairment^19^ but it depends on NO release^6^ and PP2B activation^10^. When PFs are stimulated at high frequency, long term potentiation requires MLIs activation and it is dependent on ionotropic GABA receptors and mGluR1 activation which cooperate to ensure a reliable activation of low threshold voltage-gated calcium channels. We therefore describe here a complementary pathway regulating PF to PN synaptic efficacy in a context of high frequency GCs inputs. Since GCs inputs recorded *in vivo* range from few to several hundred hertz^27^, both mechanisms might coexist in order to control plasticity in different conditions or they might underlie specific pathways expressed in different groups of GC to PN synapses as suggested by the discovery that zebrin band specific physiological mechanisms could regulate cerebellar information processing^60^,^61^.

Suggestions on the possible role played by MLI_dep_-LTP in cerebellar physiology come from previous findings obtained *in vivo*. When PFs in the cat forelimb movements-related C3 zone are electrically stimulated with the same paradigm used in this paper, a bidirectional change in PFs receptive field of PNs is induced depending on the co-activation of CF input to the recorded cell. While co-activation leads to depression, an enlargement of PFs receptive field is observed when PFs are the sole excitatory pathway stimulated. Following PFs stimulation, PNs in the C3 zone are driven by cutaneous stimulation from several parts of the body in agreement with what could be expected following awakening of silent connection between PFs and PNs^62^. In attempt to mimic protocols performed *in vivo*, LTP has been established in this study without pharmacological perturbation of synaptic transmission and intracellular signal transductions. Thus, our work might provide a detailed description of the initial molecular events which lead to the PFs receptor field enlargement observed *in vivo*. Consequently, it is tempting to speculate that the described MLI-dependent LTP could be the result of the summation of newly awaken PF to PN synapses rather than the sole increased membrane expression of AMPA receptors at activated synapses.

## Methods

### Ethical approval

All animal procedures were performed in accordance with the University of Strasbourg animal care committee’s regulations and they were approved by the Ethical Committee of the University of Strasbourg (A67-2018-38).

### Electrophysiology

Patch clamp experiments were conducted on acute coronal slices from cerebellum of adult C57BL/6 male mice (P27-P46) and age-matched Ca_V_3.1 knockout (KO) male mice from homozygous breeding. Mice were anesthetized by exposure to isoflurane, decapitated and the cerebellum dissected in ice cold bubbled (95% O_2_/5% CO_2_) aCSF containing (in mM): NaCl 120, KCl 3, NaHCO_3_ 26, NaH_2_PO_4_ 1.25, CaCl_2_ 2, MgCl_2_ 1 and glucose 16. 300 μm thick slices were obtained with a vibratome (Microm 650 V: Thermo Scientific Microm, Waltham, Massachusetts) in ice cold slicing medium containing (in mM): KGluconate 130, KCl 14.6, EGTA 2, HEPES 20, Glucose 25, pH 7.3 and supplemented with D-APV 50 μM and minocycline 50 nM. Recovery for 1–5 seconds at 35 °C was allowed in bubbled transfer buffer containing (in mM): sucrose 230, KCl 2.5, NaHCO_3_ 26, NaH_2_PO_4_ 1.25, CaCl_2_ 0.8, MgCl_2_ 8, glucose 25 and supplemented with D-APV 50 μM and minocycline 50 nM. Slices were then transferred to a holding chamber containing bubbled aCSF and kept at 35 °C for at least 40 minutes before they were moved to room temperature for the remaining experimental time.

For patch clamp recordings slices were moved to a recording chamber at 34 °C and continuously perfused with bubbled aCSF eventually supplemented with antagonists as stated in the main text. Borosilicate glass pipettes were pulled using a vertical puller (Narishige PC-10: Narishige, Tokyo, Japan) to a final resistance of 4–4.5 MΩ and filled with the following internal solution (in mM): KGluconate 130, KCl 10, MgCl_2_ 1, HEPES 10, Na_2_ATP 4, NaGTP 0.4, sucrose 16, pH 7.3. For experiments in low internal chloride concentration the following internal solution was used (in mM): KGluconate 136, KCl 4, MgCl_2_ 1, HEPES 10, Na_2_ATP 4, NaGTP 0.4, sucrose 16, pH 7.3. Once whole-cell configuration was established, a period of at least 20 minutes was waited before the start of the experiment. PNs were clamped at −60 mV and PFs-induced responses elicited by electrical stimulation delivered by a patch pipette positioned in the molecular layer distant from the recorded cell. The stimulation pipette was filled with the following solution (in mM): NaCl 120, KCl 3, HEPES 10, NaH_2_PO_4_ 1.25, CaCl_2_ 2, MgCl_2_ 1, glucose 10, pH 7.3. The PF-induced response was monitored over time by a test protocol of paired stimulation pulses (20 Hz) applied every 20 seconds. Three consecutive induced responses were averaged to obtain a mean trace of the evoked response per minute of recording. The induction protocol was applied in current-clamp mode with cells held at −68 mV.

Data were collected with a MultiClamp 700B (Molecular Devices, Sunnyvale, California), filtered at 2 kHz and digitized at 20 kHz. Data from each cell were normalized to the mean baseline value before cell averaging. Data are expressed as mean ± SEM.

A small hyperpolarize step (−10 mV) was applied before each stimulation to follow series resistance during the experiment. Series resistance was compensated by 70–80% and cells were discarded if significant changes were detected.

For input/output curves, cerebellar coronal acute slices from adult C57BL/6 and Ca_V_3.1 KO mice were prepared as previously described and the following intracellular recording solution was used in whole cell patch clamp experiments (in mM): cesium methanesulfonate 135, NaCl 6, MgCl_2_ 1, HEPES 10, MgATP 4, Na_2_GTP 0.4, EGTA 1.5, QX314Cl 5, pH 7.3. The glutamatergic transmission was kept intact during the recordings to allow the full recruitment of all MLIs contributing to the GABA-mediated inhibitory response; to minimize the EPSCs contribution to the induced response, the recorded PNs was clamped at −10 mV and the PF-mediated inhibitory response was elicited by molecular layer electrical stimulation; the stimulation electrode was pulled with a vertical puller (Narishige PC-10) to a final 5 MΩ resistance when filled with the following solution (in mM): NaCl 120, KCl 3, HEPES 10, NaH_2_PO_4_ 1.25, CaCl_2_ 2, MgCl_2_ 1, glucose 10, pH 7.3. Input/output curves were obtained by progressively increasing the strength of stimulation by 0.1 mA increments. At each intensity, PFs-mediated responses were recorded for three consecutive stimuli (one every 10 seconds) and traces averaged before analysis.

IPSPs were recorded in current clamp mode in presence of NBQX 5 μM. For IPSP/Vm curves, PNs were held at −40 mV and the membrane potential progressively hyperpolarized by 22 steps with a 200 pA increment.

### Calcium imaging

300 μm sagittal slices were prepared from adult (4–5 weeks) C57BL/6 and Ca_V_3.1 KO male mice as previously described and calcium imaging experiments were performed at room temperature. The calcium indicator Oregon Green 488 BAPTA 6F (K_D_ = ~3 μM, Molecular Probes) and heparin were added to the low chloride or regular internal solution to a final concentration of 400 μM and 50 μg/ml respectively and they were loaded into PNs via the patch clamp pipette. After whole cell establishment, the calcium indicator was allowed to diffuse for at least 20 minutes before starting the experiment. PNs where held at a potential close to −70 mV in current clamp configuration and PF stimulation was achieved by electrical stimulation via a glass stimulation pipette placed in the molecular layer. A single 100 Hz burst stimulation (15 pulses) was applied while calcium images were acquired every 50 ms by a sCMOS CCD camera (Xyla 5.5, Andor Technology Ltd, UK) with a 20 ms exposure time. Stimulation was repeated at long interval (at least 3 minutes between trials) to avoid plasticity in the recorded cell and collected images analyzed by using ImageJ^63^. Relative change in fluorescence (ΔF/F) was quantified in ROIs including the entire dendritic tree area in which PF-induced increase in calcium concentration was detected. Average ΔF/F value for each cell was obtained from 5 consecutive imaging sections before and after TTA-P2 500 nM bath application with or without SR95531 5 μM.

### Statistics

For plasticity experiments, PF-induced responses and paired-pulse ratios obtained at different time point were compared by one-way repeated measure (RM) ANOVA followed by Tukey’s post hoc test.

## Additional Information

**How to cite this article**: Binda, F. *et al.* Inhibition promotes long-term potentiation at cerebellar excitatory synapses. *Sci. Rep.* **6**, 33561; doi: 10.1038/srep33561 (2016).

## Supplementary Material

Supplementary Information
