# Supplementary material for: Inhibition promotes long-term potentiation at cerebellar excitatory synapses
Source: Sci Rep. 2016 Sep 19;6:33561. doi: 10.1038/srep33561 (PMC5027531; doi:10.1038/srep33561)
Supplement: Supplementary Information [file srep33561-s1.pdf]

**Supplementary Information**

# Inhibition promotes long term potentiation at cerebellar excitatory synapses

F. Binda*[1], K. Dorgans, S. Reibel[2], K. Sakimura[3], M. Kano[4], B. Poulain[1] and P. Isope[1]

## Methods

### Bicuculline-insensitive IPSCs recording

Cerebellar coronal acute slices from adult wild type (WT) mice were prepared as described in the main text and the following intracellular recording solution was used in whole cell patch clamp experiments (in mM): cesium methanesulfonate 135, NaCl 6, $MgCl_2$ 1, HEPES 10, MgATP 4, $Na_2GTP$ 0.4, EGTA 1.5, QX314Cl 5, pH 7.3. PNs were voltage-clamped at 0 mV and PFs-mediated inhibitory response was elicited by molecular layer electrical stimulation (the stimulation electrode was filled with the following solution, in mM: NaCl 120, KCl 3, HEPES 10, $NaH_2PO_4$ 1.25, $CaCl_2$ 2, $MgCl_2$ 1, glucose 10, pH 7.3.) in control condition and following bath application of bicuculline 20 µM and SR95531 5 µM (with bicuculline still present in the bath).

Under each experimental condition, the average trace for the PF-induced response was obtained and the baseline mean value (measured before the stimulation) subtracted. Before analysis, the total IPSC and bicuculline-insensitive IPSC for each cell were isolated by subtraction of the average trace recorded in presence of bicuculline 20 µM and SR95531 5 µM from the average trace recorded under control condition and in presence of bicuculline 20 µM respectively.

## Figure Legend

### Supplementary Figure 1: Bicuculline insensitive IPSCs component

PFs-mediated inhibitory responses from a representative PN are shown in panel a; bath application of bicuculline 20 µM strongly reduces (red trace) but it doesn’t eliminate the

IPSC recorded in the cell under control condition (black trace); the residual bicuculline-insensitive component is completely abolished by bath application of SR95531 5 µM (blue trace). Since the glutamatergic transmission is kept intact during the recordings, IPSCs are isolated by subtraction of the average trace obtained in the presence of bicuculline and SR95531 from traces recorded under control condition or in the presence of bicuculline; the integral of the isolated IPSCs was calculated for IPSQs quantification shown in panel b; bars (IPSQ = 6340.6 ± 2779.5 fC, Bic-insensitive = 383.1 ± 169.2 fC, mean ± SEM, N = 2) show the total IPSQs recorded under control condition (black) and following bicuculline bath application (red).

Bath application of bicuculline had no effect on $MLI_{dep}$-LTP induced in low intracellular chloride as shown in panel c and quantified in panel d (N = 5, paired t-test, P = 0.015).

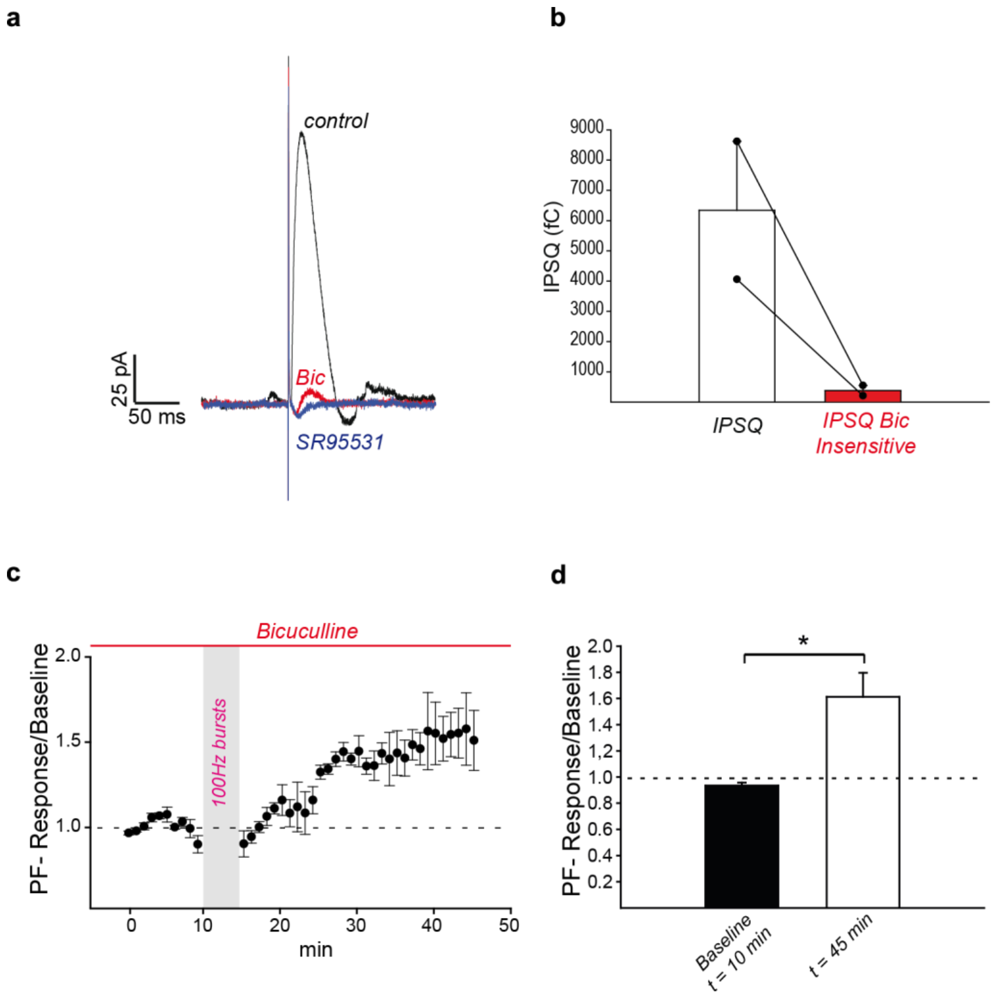
a
control
Bic
SR95531
25 pA
50 ms
b
IPSQ (fC)
9000
8000
7000
6000
5000
4000
3000
2000
1000
IPSQ
IPSQ Bic Insensitive
c
Bicuculline
100Hz bursts
PF- Response/Baseline
2.0
1.5
1.0
0
10
20
30
40
50
min
d
*
PF- Response/Baseline
2.0
1.8
1.6
1.4
1,2
1.0
0.8
0.6
0.4
0.2
Baseline t = 10 min
t = 45 min
